# Supplementary material for: Assessment of cognitive functioning after living kidney donation: A cross-sectional pilot study
Source: PLoS One. 2022 Feb 25;17(2):e0264284. doi: 10.1371/journal.pone.0264284 (PMC8880950; doi:10.1371/journal.pone.0264284)
Supplement: S1 File — (DOCX) [file pone.0264284.s001.docx]

**Neurokid_data**

Number – Study number

Age – Age at time of assessment (metric)

Gender – Gender (categorial)

Recipient – Recipient of the organ (categorial)

1. partner
2. parent
3. child
4. brother/sister
5. aunt/uncle/cousin
6. Brother/sister-in-law
7. friend
8. grandchild
9. nephew/niece

Group – study group (binary, 1=Donors, 2=controls)

Timesincedonation_months – time since donation in months (metric)

Education_yrs – education in years (metric)

Ageatdonation – Age of donors at time of donation (metric)

Hypertonus – presence of hypertonus (binary, 1=yes, 0=no)

KHK – presence of coronary heart disease (binary, 1=yes, 0=no)

Hypothyreosis – presence of hyperthyreosis (binary, 1=yes, 0=no)

DM – presence of diabetes mellitus (binary, 1=yes, 0=no)

Hyperlip – presence of Hyperlipidemia (binary, 1=yes, 0=no)

WFMT_W_RV – Word figure memory test, word, raw value (metric)

WFMT_W_path - Word figure memory test, word (binary, 1=abnormal, 0=normal)

WFMT_F_RV - Word figure memory test, figure, raw value (metric)

WFMT_F_path - Word figure memory test, figure (binary, 1=abnormal, 0=normal)

RF_Nons_RV – Recurring figures test, nonsense, raw value (metric)

RF_Nons_path - Recurring figures test, nonsense (binary, 1=abnormal, 0=normal)

RF_Geom_RV - Recurring figures test, geometric, raw value (metric)

RF_Geom_path - Recurring figures test, geometric (binary, 1=abnormal, 0=normal)

D2_Fpercent_RV – D2 test, errors (%), raw value (metric)

D2_Fpercent_Score - D2 test, errors (binary, 1=abnormal, 0=normal)

D2_summinF_RV – D2 test, Error-corrected total number, raw value (metric)

D2_summinF_Score – D2 test, error-corrected total number (binary, 1=abnormal, 0=normal)

D2_CC_RV – D2 test, capacity of concentration, raw value (metric)

D2_CC_Score – D2, capacity of concentration (binary, 1=abnormal, 0=normal)

TAP_Al_wCue_RV – TAP, Alertness, with cue, raw value (ms, metric)

TAP_Al_wCue_Score – TAP, Alertness, with cue (binary, 1=abnormal, 0=normal)

TAP_Al_woCue_RV – TAP, Alertness, without cue, raw value (ms, matric)

TAP_Al_woCue_Score – TAP Alertness, without cue (binary, 1=abnormal, 0=normal)

TAP_WM_MD_RV – TAP, Working memory, median, raw value (ms, metric)

TAP_WM_MD_Score - TAP, Working memory, median (binary, 1=abnormal, 0=normal)

TAP_WM_errors_RV – TAP, Working memory, errors, raw value (ms, metric)

TAP_WM_errors_Score – TAP, Working memory, errors (binary, 1=abnormal, 0=normal)

TAP_WM_Miss_RV – TAP, Working memory, misses, raw value (metric)

TAP_WM_Miss_Score – TAP, Working memory, misses (binary, 1=abnormal, 0=normal)

TAP_Cross_MD_RV – TAP, Crossmodal integration, median, raw value (ms, metric)

TAP_Cross_MD_Score – TAP, Crossmodal integration, median (binary, 1=abnormal, 0=normal)

TAP_Cross_errors_RV – TAP, Crossmodal integration, errors (metric)

TAP_Cross_errors_Score – TAP, Crossmodal integration, errors (binary, 1=abnormal, 0=normal)

TAP_Flexi_MD_RV – TAP, Flexibility, median, raw value (ms, metric)

TAP_Flexi_MD_Score – TAP, Flexibility, median (binary, 1=abnormal, 0=normal)

TAP_Flexi_errors_RV – TAP, Flexibility, errors (metric)

TAP_Flexi_errors_Score – TAP, Flexibility, errors (binary, 1=abnormal, 0=normal)

TAP_DA_MDaud_RV – TAP, Divided attention, median auditive, raw value (ms, metric)

TAP_DA_MDaud_Score – TAP, Divided attention, median auditive (binary, 1=abnormal, 0=normal)

TAP_DA_MDvis_RV – TAP, Divided attention, median visual, raw value (ms, metric)

TAP_DA_MDvis_Score – TAP, Divided attention, median visual(binary, 1=abnormal, 0=normal)

TAP_DA_errors_RV – TAP, Divided attention, errors (metric)

TAP_DA_errors_Score – TAP, Divided attention, errors (binary, 1=abnormal, 0=normal)

TAP_DA_Miss_RV – TAP, Divided attention, misses, raw value (metric)

TAP_DA_Miss_Score – TAP, Divided attention, misses (binary, 1=abnormal, 0=normal)

TAP_Go_MD_RV – TAP Go/No Go, median, raw value (ms, metric)

TAP_Go_MD_Score – TAP Go/No Go, median (binary, 1=abnormal, 0=normal)

TAP_Go_errors_RV – TAP Go/No Go, errors, raw value (metric)

TAP_Go_errors_Score – TAP Go/No Go, errors (binary, 1=abnormal, 0=normal)

TAP_I_MD_RV – TAP, Incompability, median, raw value (ms, metric)

TAP_I_MD_Score – TAP, Incompability, median (binary, 1=abnormal, 0=normal)

TAP_I_errors_RV – TAP, Incompability, errors, raw value (metric)

TAP_I_errors_Score – TAP, Incompability, errors (binary, 1=abnormal, 0=normal)

TAP_VA_vLi_MD_RV – TAP, covert shift of attention, valid left, median, raw value (ms, metric)

TAP_VA_vLi_MD_Score – TAP, covert shift of attention, valid left, median (binary, 1=abnormal, 0=normal)

TAP_VA_vRe_MD_RV – TAP, covert shift of attention, valid right, median, raw value (ms, metric)

TAP_VA_vRe_MD_Score – TAP, covert shift of attention, valid right, median (binary, 1=abnormal, 0=normal)

TAP_VA_ivLi_RV – TAP, covert shift of attention, invalid left, median, raw value (ms, metric)

TAP_VA_ivLi_Score – TAP, covert shift of attention, invalid left, median (binary, 1=abnormal, 0=normal)

TAP_VA_ivRe_RV – TAP, covert shift of attention, invalid right, median, raw value (ms, metric)

TAP_VA_ivRe_Score – TAP, covert shift of attention, invalid right, median (binary, 1=abnormal, 0=normal)

AttSumScore – Attentiontest sumscore (metric)

AttSumScore_path – Attentiontest sumscore (binary, 1=abnormal, 0=normal)

eGFR_preop – eGFR pre-operation (metric)

eGFR_postop – eGFR post-operation (metric)

eGFR_firstvisit – eGFR at first visit (metric)

eGFR_lastvisit – eGFR last visit (metric)

PSCS12 – Physical subscale of SF-12 (metric)

MSC12 – Mental subscale of SF-12 (metric)

GSI – Global severity index of SCL-9 (metric)

MFI_Gen – MFI subscale general (metric)

MFI_Phys – MFI subscale physical (metric)

MFI_Aktiv – MFI subscale activity (metric)

MFI_Motiv – MFI subscale motivation (metric)

MFI_Mental – MFI Subscale mental (metric)

GADtotal – GAD (metric)

PHQtotal – PHQ (metric)
