# Supplementary material for: Assessment of cognitive functioning after living kidney donation: A cross-sectional pilot study
Source: PLoS One. 2022 Feb 25;17(2):e0264284. doi: 10.1371/journal.pone.0264284 (PMC8880950; doi:10.1371/journal.pone.0264284)
Supplement: S2 File — (DOCX) [file pone.0264284.s002.docx]

Neurokid Inductive

Number – Study number

Age – Age at time of assessment (metric)

Gender – Gender (categorial)

Recipient – Recipient of the organ (categorial)

1. partner
2. parent
3. child
4. brother/sister
5. aunt/uncle/cousin
6. Brother/sister-in-law
7. friend
8. grandchild
9. nephew/niece

Group – study group (binary, 1=Donors with cognitive assessment, 2=donors only questionnaire)

Timesincedonation_years– time since donation in years(metric)

Education_yrs – education in years (metric)

Ageatdonation – Age of donors at time of donation (metric)

PSCS12 – Physical subscale of SF-12 (metric)

MSC12 – Mental subscale of SF-12 (metric)

GSI – Global severity index of SCL-9 (metric)

MFI_Gen – MFI subscale general (metric)

MFI_Phys – MFI subscale physical (metric)

MFI_Aktiv – MFI subscale activity (metric)

MFI_Motiv – MFI subscale motivation (metric)

MFI_Mental – MFI Subscale mental (metric)

GADtotal – GAD (metric)

PHQtotal – PHQ (metric)
