# Supplementary material for: Assessment of cognitive functioning after living kidney donation: A cross-sectional pilot study
Source: PLoS One. 2022 Feb 25;17(2):e0264284. doi: 10.1371/journal.pone.0264284 (PMC8880950; doi:10.1371/journal.pone.0264284)
Supplement: Attachment — Submitted filename: Response to reviewers Neurokid.docx [file pone.0264284.s005.docx]

**Point-by-point response to reviewers comments and questions**

**PONE-D-21-31779**

**Assessment of cognitive functioning after living kidney donation: a cross-sectional pilot study**

**Reviewer #1**

I have a few points, which I feel require further clarification:

1. Was there a reason why the quality of life measures were not given to the control group?

*The main quality of life instrument, the SF-12 (with the subscales PCS and MCS), was actually completed also by the controls (see Table 1). We found no difference between groups and both groups showed scores within the normal range for quality of life. Only the living donors filled out the other self-rating measures (fatigue, depressive and anxiety symptoms) as part of a more comprehensive assessment battery within the follow-up study.*

2. Did you do a sample size calculation?

*This investigation was designed as a pilot study. Since this is the first study investigating cognitive functioning in individuals after living kidney donation, we did not have a solid database for a power calculation.*

3. Why were patients with mental health problems excluded?

*Mental disorders such as depression can affect cognitive performance. We wanted to eliminate one strong potentially confounding factor. As mentioned in the discussion, “Depression is known to affect cognitive functioning”. We supported this statement by citing two references:*

*39. Ahern E, Semkovska M. Cognitive functioning in the first-episode of major depressive disorder: a systematic review and meta-analysis. Neuropsychology. 2017;31:52–72.*

*40. Liu J, Liu B, Wang M, Ju Y, Dong Q, Lu X, et al. Evidence for progressive cognitive deficits in patients with major depressive disorder. Front Psychiatry. 2021;12:627695.*

I have minor comments re the language:

*We thank the reviewer for carefully reading and editing the manuscript. We followed the suggested changes.*

Line 50: the term ‘deceased donation’ is considered more appropriate than post-mortem donation.

*We agree with the reviewer’s suggestion and substituted “post-mortem” with “deceased”.*

Line 51: I wouldn’t call rehospitalisation a side-effect – it is a complication

*We changed the wording accordingly.*

Line 53: Long run ought to be long term

*We changed “long run” to “long term”.*

Line 56-59: I am unsure of what is meant by “A meta-analysis could show…”

*We re-phrased the sentence: “A systematic review revealed that most, but not all, cross-sectional and longitudinal studies suggest an association between cognitive impairment and CKD.”*

Line 154: There is a typo – der instead of the

*We corrected the typo. Thanks for catching that.*

Line 244: I would remove the word ‘group’

*We omitted the word “group”*

Line 253-258: These three sentences are written poorly and are confusing in what they are trying to say. Please rephrase these.

*The sentences were re-written and readability should be improved.*

*“Regarding the pattern of abnormal cognitive test results, we found similarities and differences between our sample of LKD and individuals with CKD in general population samples. General and visual attention and concentration was impaired in the LKD in our study similarly to individuals with CKD in population samples. However, in contrast to the LKD in our study, individuals with CKD frequently also exhibit memory impairment [10-12, 34].”*

**Reviewer #2**

This is a thorough and diligently performed study, well designed, with a clear research questions, clearly and accurately written. I have no comments for improvement. The study can be published as it is. If I would have a minor comment, it would refer to how the authors explain the reduced scores in working memory, slower reaction times and divided attention, meaning how do they explain the relationship between eGFR and these low scores... Do they assume it is a physical parameter making the difference? Or could it be that living kidney donors have an additional "worry" as in an affective rumination going on permanently related to the loss of their kidney and a potential risk for kidney function due to donation that affects working memory? Please consider this maybe unorthodox idea more of a comment in terms of an informal academic exchange rather than a comment for revision or improvement.

*We thank the reviewer for his/her supporting comments. It might be possible that “worries” influence cognitive performance; however, in our experience with working with living donors this is not a frequent problem. In addition, as can be seen in Table 1, quality of life, depression, anxiety, fatigue and levels of distress were within normal ranges.*
